# Physiological and molecular mechanisms of the response of roots of *Pinus massoniana* Lamb. to low-temperature stress

**DOI:** 10.3389/fpls.2022.954324

**Published:** 2022-09-28

**Authors:** Jingyu Lu, Hu Chen, Zhangqi Yang, Shuang Sun, Qunfeng Luo, Junkang Xie, Jianhui Tan

**Affiliations:** ^1^ Key Laboratory of Central South Fast-Growing Timber Cultivation of Forestry Ministry of China, Guangxi Forestry Research Institute, Nanning, China; ^2^ Guangxi Key Laboratory of Superior Timber Trees Resource Cultivation, Nanning, China; ^3^ Masson Pine Engineering Research Center of the State Forestry Administration, Nanning, China; ^4^ Masson Pine Engineering Research Center of Guangxi, Nanning, China

**Keywords:** *Pinus massoniana* Lamb., low-temperature stress, physiology and biochemistry, transcriptomics, cold resistance gene

## Abstract

*Pinus massoniana* Lamb. is the timber species with the widest distribution and the largest afforestation area in China, providing a large amount of timber, turpentine and ecological products. but low temperature limits its growth and geographical distribution. Physiological and molecular studies can well explain the mechanism of *P. massoniana* response to low temperature. In this study, physiological and biochemical indexes, cell morphology, lignin content, gene regulatory networks, and gene expression patterns of different *P. massoniana* varieties (cold-tolerant and cold-sensitive) were studied from physiological, biochemical, and molecular perspectives. The results indicated that under low-temperature stress, the cold-tolerant cultivar maintained high contents of osmoregulatory substances, and the root morphology and structure remained intact. In the initial stage of low-temperature stress, the number of differentially expressed genes was 7148, and with the extension of stress time, the number of differentially expressed genes decreased to 1991. P*. massoniana* might direct its responses to low temperature by regulating phenylpropane metabolism, starch and sucrose metabolism, hormone signaling pathways, and transcription factors. *BAM, 4CL, CCoAOMT, PRX5, WRKYs*, and hormone synthesis related genes play important roles. *P. massoniana* cultivars may vary in response mechanisms. In this study, physiological and analytical techniques were used to study the root tip response mechanism of Masson’s pine to low temperature stress. The results of this study lay a foundation for in-depth research on the molecular functions of *P. massoniana* under low-temperature stress conditions.

## Introduction

Low temperature is an important ecological factor that limits the growth and geographical distribution of plants, and severe low-temperature stress leads to plant death (Shinozaki, 2000; Knight and Knight, 2001; Mccully et al., 2004; Jiang et al., 2020). In recent years, researchers have conducted many studies of the morphology, tissue structure, cell membrane systems, protective enzyme systems, physiological metabolism, gene regulatory networks, and gene functions of plants maintained at low temperatures (Zhang et al., 2019; Ding et al., 2019).

When plants resist low-temperature stress, their plasma membranes, antioxidant enzyme systems, osmoregulatory substances, and endogenous hormones produce stress responses that are used for defense through a series of complex regulatory mechanisms. Low-temperature stress causes plants to activate antioxidant enzyme systems that scavenge reactive oxygen species (ROS) (Nasef, 2018; Sigaud-Kutner et al., 2010; Li et al., 2018). This activation increases production of osmoregulatory substances, such as proline, soluble sugars, and soluble protein. These reactions endow plants with a variety of osmoregulatory activities, thereby improving their cold resistance (Hare et al., 2010; Zhang et al., 2018). In addition, cultivars with strong cold resistance have low cell membrane permeability, low relative conductivity, and low malondialdehyde (MDA) content (Karami-Moalem et al., 2018).

Through long-term evolution, plants have developed survival mechanisms by which they adapt to low temperatures. At the molecular level, the C-repeat (CRT)-binding factor/dehydration-responsive element (DRE) binding protein 1 (CBF/DREB1)-dependent cold signaling pathway and related transcription factors (TFs) form the core of the cold resistance response and have been extensively studied over the past two decades (Stockinger et al., 1997; Novillo et al., 2007; Jiang et al., 2020; Zhao et al., 2022). Precise regulation by TFs is conducive to the analysis and improvement of the complex regulatory signal networks induced by abiotic stress in plants (Lee and Young, 2000). Low-temperature signals activate TFs through signal transduction pathways and interact with cis-acting elements in the promoter regions of cold response-related genes, thereby activating the expression of downstream genes (Agarwal and Jha, 2010). A large number of TFs, including AP2/ERF (Lv et al., 2019), MYB (Zhu et al., 2005; Wang et al., 2019), NAC (Ohbayashi and Sugiyama, 2018; Zhuo et al., 2018), and WRKY (Rushton et al., 2012; Wang et al., 2014), have been shown to be associated with cold resistance (Kidokoro et al., 2022). Osmotic stress resistance, the antioxidant system, and hormone-regulated gene expression are key to resisting low-temperature stress, and related genes regulate the increases in contents of certain substances, thereby enhancing the tolerance of plants to low temperatures (Cuevas et al., 2009; Hare et al., 2010; Li et al., 2018; Zhang et al., 2018).

Roots are responsible for the absorption, storage, and supply of nutrients and carbohydrates in plants (Brunner and Godbold, 2007) and are key organs by which plants adapt to stress (Purushothaman et al., 2013). Low temperature leads to a decrease in the water transport rate in the root system and to a water deficit that reduces a plant’s ability to adapt to climatic changes (Bloom et al., 2004). In addition, roots can release volatile chemicals when plants are under stress, thereby affecting the surrounding environment (Quan and Ding, 2017; Zhou et al., 2019; Rich-Griffin et al., 2020).

There are few studies on the mechanisms of responses to low-temperature stress in tree species of timber-producing forests. Studies have mainly focused on a few types of trees, such as *Populus* spp. L., *Pinus sylvestris*, and *Picea abies* (Joosen et al., 2006; Maestrini et al., 2009; Benedict et al., 2010; Vergara et al., 2022), and have addressed antioxidant enzymes (Renaut et al., 2006) and TFs (Vergara et al., 2022). Recently, researchers have paid more attention to the relationship between cold resistance and lignin and starch synthesis pathways (Zhao et al., 2019; Liang et al., 2022; Liu et al., 2022).

*Pinus massoniana* (Pinaceae) is a gymnosperm with the characteristics of rapid growth, high yield, a high degree of comprehensive utilization, and high economic value and the most important high-quality coniferous timber species in southern China (Yang et al., 2020). Due to global climate change and the frequent occurrence of extreme weather events over the last 20 years, trees worldwide have suffered frost damage and death due to low temperatures. In the area where *P. massoniana* is produced, several rare low-temperature disasters have occurred, resulting in massive tree mortality and a 39% decrease in retention of forest stands, but only a small number of studies of cold resistance in *P. massoniana* at the hormonal and physiological levels have been conducted (Yang, 2011). No studies have reported the effects of low-temperature stress on the physiological and biochemical properties, microstructure, and expression of cold resistance genes in *P. massoniana* roots. Therefore, this study investigated physiological indexes, microstructure, lignin content, regulatory gene networks, and key genes in cultivars differing in cold resistance under low-temperature conditions to provide a basis for in-depth studies of the physiological and molecular mechanisms employed by *P. massoniana* in response to low temperatures.

## Materials and methods

### Materials

In this study, two different *P. massoniana* cultivars, GC0209D (cold tolerant, abbreviated T) and GC0265D (cold sensitive, designated S), which were previously obtained from a second-generation clonal seed orchard of *P. massoniana*, were used as experimental materials. We obtained permission to collect samples from the two germplasm strains. The experimental research and field studies on plants complied with relevant institutional, national, and international guidelines and legislation. Plump seeds (seed germination rate reached 98%) of the two cultivars were germinated after sterilization, in which seeds were sterilized with a solution of potassium permanganate (1:3500 of volume fraction) for 10 min, cleaned with sterile water three to five times for 1 min each, and then soaked with water at 45°C for 1 d. These seeds were evenly distributed in a sterilized yellow soil tray, covered with yellow soil, and then drenched with water. Seedlings were cultured in seedling beds (conditions were held constant with an artificial climate box). After cotyledons emerged from the hull, seedlings were transferred to larger cups (12 cm wide and 20 cm high) containing loess obtained from orchards under *P. massoniana* trees. Seedlings were placed in an artificial climate chamber (temperature: 27°C; humidity: 60%; day/night: 16 h/8 h light cycle, light intensity set to 1200 lx) (Tan, 2013) and allowed to grow for 30 d. Then, seedlings of uniform size were selected and placed in a climate chamber for low-temperature treatment.

### Low-temperature treatment

Seedlings of each variety were divided into six groups; there were three pots in each group and 60 plants in each pot. All pots were placed in an artificial cold climate chamber at 25°C for 3 d prior to the stress treatment. With plants maintained at 25°C as the control, low-temperature stress treatments of 10°C, 0°C, and -5°C were performed for 24 h. Some of the plants that had been subjected to the 10°C and 0°C treatments were then placed in a 25°C incubator and allowed to recover for 24 h (heating rate: Li et al., 20184°C/h); these are referred to as 10°C R and 0°C R, respectively. At the end of each treatment, the plants’ roots (sections 3 cm in length taken from the tips of the main root and the lateral roots) were used for the determination of physiological and biochemical indexes, lignin content, starch content, and gene expression. Based on the morphological, physiological, and biochemical results, root samples from plants of the S cultivar that had been exposed to a temperature of 0°C for 0 h (control), 24 h (L1), and 48 h (L2) were selected and stored in liquid nitrogen for transcriptome sequencing.

### Determination of physiological indexes

Relative conductivity was measured using a conductivity meter (DDS-11A, Leici, Shanghai, China). We washed the samples three times with ultrapure water and dried them using clean filter paper. We cut root samples into 0.5-cm segments using clean scissors and placed them into 50-mL centrifuge tubes; there were three biological replicates for each treatment. After an additional 25 mL of ultrapure water was added, the tubes were centrifuged under vacuum for 30 min at room temperature and then allowed to stand for 20 min. The initial conductivity (R1) of the samples was measured at room temperature. The centrifuge tubes with samples were then placed in a boiling water bath for 20 min; after the contents had reached room temperature, the conductivity after boiling (R2) was measured. The relative conductivity was calculated as (R1/R2)×100% (Sun et al., 2020).

Each sample to be tested was placed into a mortar and ground into powder with liquid nitrogen. Then, 0.1 g was weighed. The contents and activities of superoxide dismutase (SOD), peroxidase (POD), proline, MDA, soluble sugars, and soluble proteins were determined using the nitroblue tetrazolium (NBT) method, guaiacol method, ninhydrin colorimetric method, thiobarbituric acid colorimetry, anthrone colorimetric method, and bicinchoninic acid (BCA) method, respectively. In these tests, reagent kits were from Suzhou Keming Biotechnology Co., Ltd. (Suzhou, China). The optical densities (ODs) of the samples were measured using a SuPerMax 3100 microplate reader (Shanghai, China), with three repeats per sample.

We used paraffin sections for microstructural observations. Fast green-iodine-potassium iodide staining was used to observe starch content. Phloroglucinol staining was used to observe changes in lignin content (Guo et al., 2021). An EG 1150H embedding station (Leica, Germany), an RM2235 automatic microtome (Leica, Germany), and a DS-Ri2 microscope (Nikon, Japan) were used.

### Transcriptome sequencing

For the 0°C treatment, sampling timepoints of 0 h (CK), 24 h (L1), and 48 h (L2) were used, and three biological replicates were performed for each treatment, resulting in a total of nine samples (CK1, CK2, and CK3; L1-1, L1-2, and L1-3; L2-1, L2-2, and L2-3). Total RNA was extracted, and an RNA-seq library was constructed. The RNA extraction kit was from Tiangen Biotech Co., Ltd. (Beijing, China), and the reverse transcription kit was from Bao Biological Engineering Co., Ltd. (Dalian, China); the specific methods are in the instructions provided with the kits. BGI Biotechnology Co., Ltd. (Shenzhen, China) performed transcriptome sequencing.

The raw data were subjected to quality control to eliminate low-quality data and obtain clean reads. The third-generation full-length transcriptome of *P. massoniana* was used as the reference genome for subsequent analyses, including gene quantitative analysis, gene expression-based principal component analysis (PCA), and correlation analysis. Differentially expressed gene (DEG) screening, Gene Ontology (GO) and Kyoto Encyclopedia of Genes and Genomes (KEGG) pathway analyses of DEGs, gene clustering, and key gene mining were performed. Analysis of DEGs was performed using the DESeq R software package (Love et al., 2014), and p < 0.01 and log_2_(|fold change|) ≥ 2 were used as the criteria.

### Analysis of gene expression patterns

In this study, we referred to the relevant literature (Zhang et al., 2016; Liu et al., 2018; Lv et al., 2019; Liang et al., 2022; Liu et al., 2022). We focused on the pathways with the largest numbers of genes differentially expressed in response to low-temperature stress in *P. massoniana*, such as the phenylpropanoid biosynthesis pathway, starch and sucrose metabolism pathway, plant hormone signal transduction pathway, and AP2/ERF (Lv et al., 2019) and WRKY (Rushton et al., 2012; Wang et al., 2014) transcription factors were discovered plants responding more TFs to abiotic stress. We selected the three genes with the highest fold change of AP2/ERF and WRKY transcription factor genes in low temperature stress, respectively (**Supplementary Figure 1**). Gene expression pattern analysis was performed using quantitative real-time polymerase chain reaction (qRT−PCR). *PmUBI4* and *PmCYP* (Chen et al., 2016) were selected as the internal reference genes, and primers (**Supplementary Table 1**) were designed using Primer 6.0 software and synthesized at Sangon Biotech Co., Ltd. (Shanghai, China). A CFX96 Touch qRT−PCR instrument (Bio-Rad, USA) was used together with a qRT−PCR kit purchased from Bao Biological Engineering Co., Ltd. Three biological replicates were performed for each sample. PCR was performed according to the instructions provided with the kit. The relative expression levels were analyzed with the 2^-ΔΔCT^ method (Livak and Schmittgen, 2002).

### Data analysis and mapping

Microsoft Excel 2010 was used for data processing, SPSS 26.0 software was used for analysis of variance (ANOVA), and the DESeq package in R software was used for DEG screening. KEGG and GO enrichment analyses were performed using the KEGG and GO dynamic data analysis tools provided by Gene *De Novo* Co. (Guangzhou, China), MapMan 3.6.0 RC1 software (https://mapman.gabipd.org/) was used for metabolic pathway mapping, and TBtools v1.087 (Chen et al., 2020) and GraphPad Prism 7.0 (GraphPad Software, San Diego, CA, USA) were used to plot gene cluster heatmaps and Venn diagrams.

## Results

### Effects of low-temperature stress on the physiological and biochemical indexes of roots

**Figure 1:**
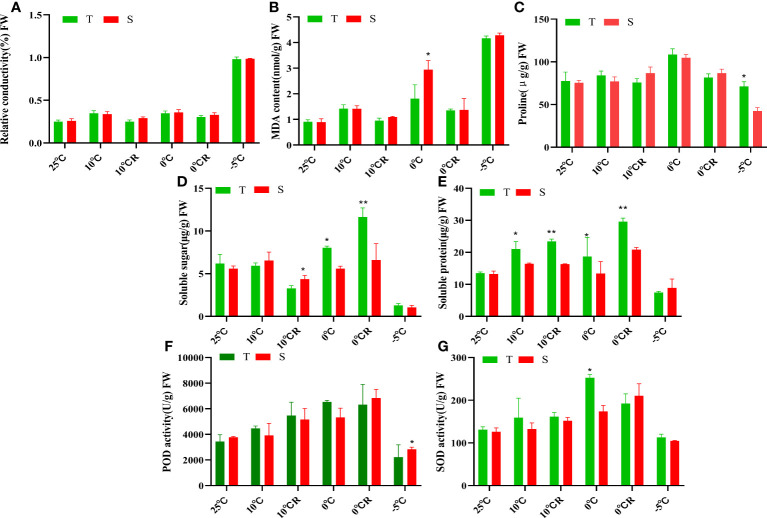
Effects of low-temperature stress on physiological indexes of roots of *P. massoniana* with different cold resistance. **(A–G)** are the relative conductivity, the MDA contents, the proline contents, the soluble sugar contents, the soluble protein content, the POD activity, and the POD activity, respectively. *P < 0.05, Student t tests, **P < 0.01, Student t tests.

The relative conductivity values of the roots of *P. massoniana* plants exposed to low-temperature stress increased with decreasing temperature. Although 0°C stress caused a certain degree of damage to the cell membranes of the plants, this damage was slowly restored in the 10°C R and 0°C R groups, while -5°C stress caused irreversible damage to cell membranes (**Figure 1A**). The MDA contents of both cultivars gradually increased with decreasing temperature; however, plants exposed to -5°C stress could no longer compensate for the damage to cell membranes caused by low temperature, resulting in elevated membrane lipid peroxidation. At 0°C, the MDA content of GC0209D was lower than that of GC0265D, indicating stronger cold resistance in the former cultivar (**Figure 1B**). The proline contents of the roots of the two cultivars increased overall in plants exposed to low-temperature stress, with the greatest accumulation in plants exposed to 0°C stress (**Figure 1C**). After exposure to low-temperature stress above 0°C, the soluble sugar content, soluble protein content, SOD activity, and peroxidase (POD) activity of GC0209D were all higher than those of the control, while the soluble sugar content of GC0265D was higher than that of the control, and its soluble protein content did not differ from that of the control. After exposure to cold stress at -5°C, the values of all three of these indexes were higher in GC0209D than in GC0265D, indicating that GC0209D maintained high contents of osmoregulatory substances under cold stress conditions to regulate cell permeability and enhance cold resistance (**Figures 1D–G**, **Supplementary Table 2**).

### Effects of low-temperature stress on root morphology and structure

**Figure 2:**
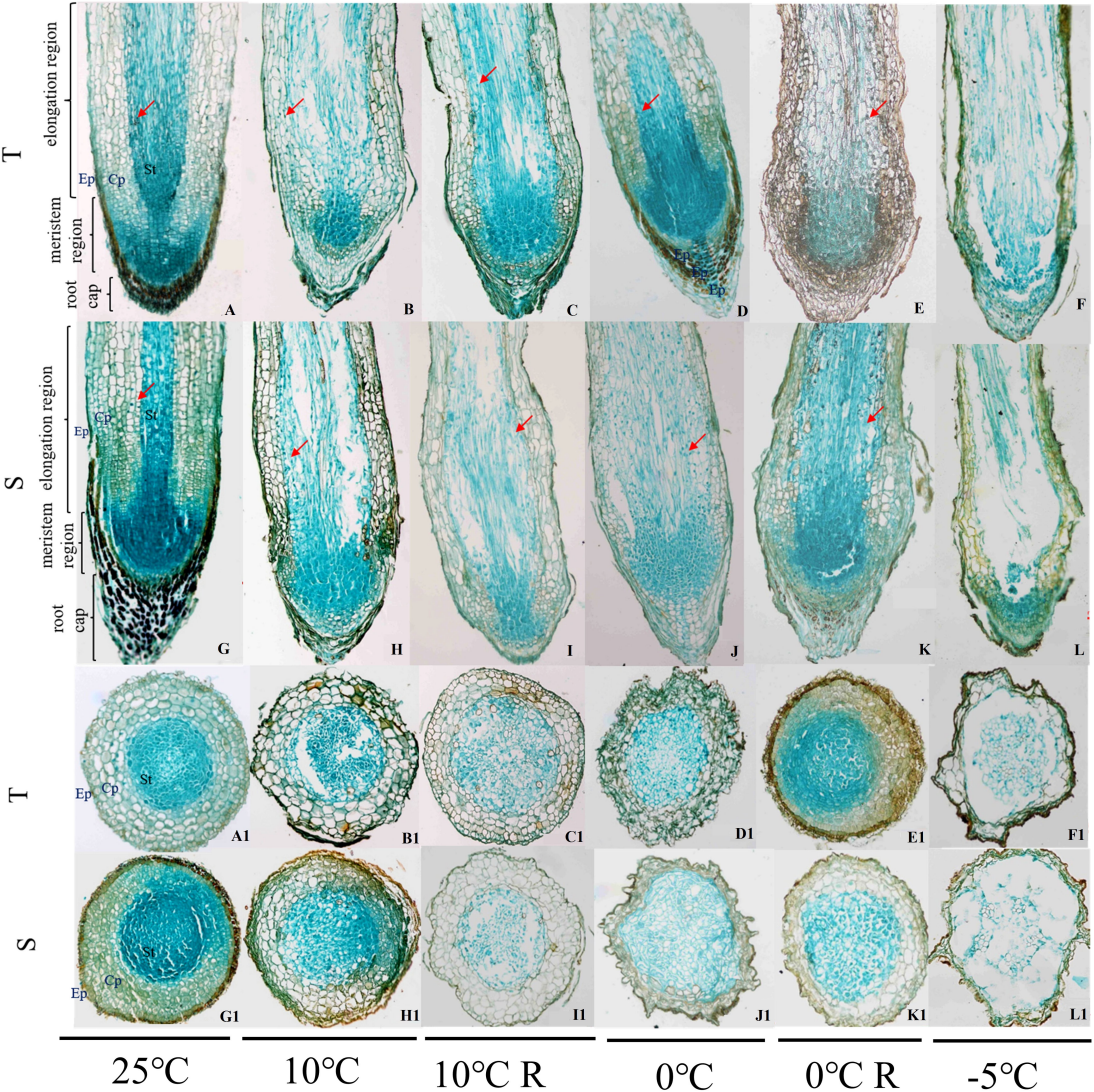
Effects of low-temperature stress on the root anatomical structure of *P. massoniana* seedlings. Ep, epidermis; Cp, cortex; St, stele. **A-F** and **A1-F1** show the longitudinal and transverse sections of roots of the cold-resistant cultivar (T) in the 25°C, 10°C, 10°C R, 0°C, 0°C R, and -5°C treatments, respectively; **G-L** and **G1-L1** are the longitudinal and transverse sections of roots of the cold-sensitive cultivar (S) in the 25°C, 10°C, 10°C R, 0°C, 0°C R, and -5°C treatment groups, respectively. The red arrow shows starch grains.

The root cap, meristematic zone, and elongation zone can be seen in longitudinal sections of the root tips of *P. massoniana*, and the epidermis, cortex, and stele can be seen in transverse sections (**Figure 2**). With decrease in temperature, the cells forming the parenchyma of the apical cortex were arranged neatly and tightly, the cell gaps in the stele were small and slightly deformed, the columellar cell gaps increased, and the cells showed a disordered arrangement; some cells were ruptured, and the arrangement of the cells in the stele was loose, with larger cell gaps. When the temperature reached -5°C, the cells collapsed and lysed; some cells were attached to each other in the epidermis and cortex, the cells in the stele were collapsed and deformed, the cells in the meristematic zone were loosely arranged and had separated from the root cap, and the cavity formed between the cells in the stele and the cortex in the elongation zone was much larger in the GC0265D cultivar than in the GC0209D cultivar (**Figure 2**). In the 10°C R group, the morphology of the cells in GC0209D was similar to that of the control, while GC0265D still exhibited cell deformation. In the 0°C R group, there was some recovery after the 0°C treatment, but some thin-walled superficial and cortical cells remained significantly deformed compared with the control, and some cells in the stele were arranged in a disorderly manner.

### Basic transcriptome data and DEG screening

**Figure 3:**
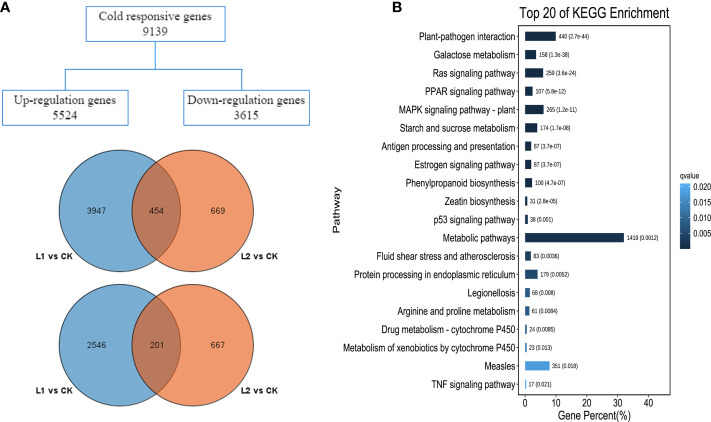
Basic results of transcriptome data analysis. **(A)** Venn diagram of differentially expressed genes, upregulated expression gene Wayne diagram (left), and downregulated expression gene Wayne diagram (right). **(B)** KEGG enrichment analysis of differentially expressed genes.

For all samples, the proportions of Q20 and Q30 were 96.36-96.78% and 86.38-90.56%, respectively, and the guanine-cytosine (GC) content ranged from 45.3-46.1%, indicating that the quality of the sequencing was good. Using the third-generation full-length transcriptome of *P. massoniana* as the reference genome, the percentage of clean reads ranged from 74.15-75.11%; because these values were greater than 70%, all the reads could be used in the subsequent analysis (**Supplementary Table 3**). The R^2^ among the three biological replicates was ≥ 0.94 (**Supplementary Figure 2**), indicating a strong correlation among the samples. The sequencing results indicated that there were 7148 DEGs at the beginning of the low-temperature stress, 4401 of which were upregulated (L1 vs. CK). As the duration of cold stress increased, the number of DEGs decreased to 1991, of which 1123 DEGs were upregulated (L2 vs. CK). There were 454 upregulated genes and 201 downregulated genes in the two groups (**Figure 3A**). GO enrichment separated the functions of the DEGs into three categories: biological processes, molecular functions and cellular components (**Supplementary Figure 3**). The top 20 significantly enriched KEGG metabolic pathways are shown in **Figure 3B**. The results showed that the number of genes enriched in metabolic pathways associated with plants was high; metabolic pathways involving plant−pathogen interactions, mitogen-activated protein kinase (MAPK) signaling, galactose metabolism, starch and sucrose metabolism, and phenylpropanoid biosynthesis showed high enrichment, indicating that these pathways may play roles in the responses of *P. massoniana* roots to low-temperature stress.

### Key metabolic pathways and cold resistance in *P. massoniana*

Based on the results of GO and KEGG analyses, this study analyzed pathways and gene expression related to phenylpropanoid metabolism, carbohydrate metabolism, TF regulation, and hormone signal transduction in *P. massoniana* seedlings in response to low-temperature stress. According to the results of KEGG enrichment analysis, the pathways with the largest numbers of enriched DEGs were related to metabolic processes. DEGs were significantly enriched in pathways related to carbohydrate metabolism and biosynthetic metabolism of other secondary metabolites; 174 and 100 DEGs were associated with the starch and sucrose metabolic pathways and the phenylpropanoid biosynthesis pathway, respectively. The results are shown in **Supplementary Figures 4**, **5**.

### Analysis of the expression of cold resistance-related genes in various tissues

**Figure 4:**
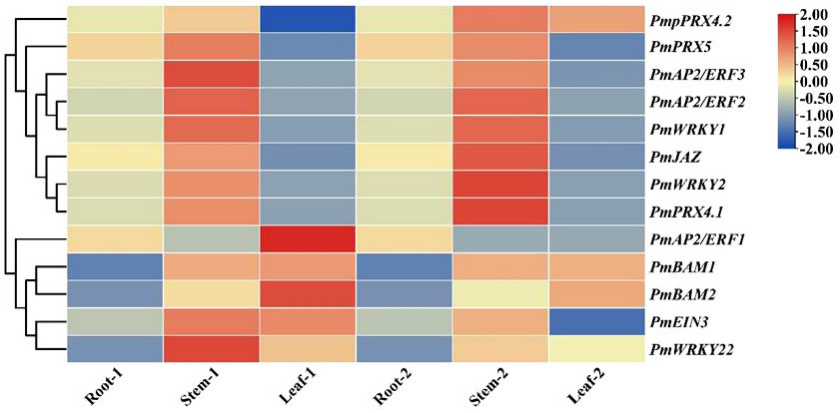
Expression of 13 candidate cold resistance genes in the roots, stems, and leaves of *P. massoniana* seedlings differing in cold resistance. Note: Root-1, Stem-1 and Leaf-1 represent the roots, stems, and leaves, respectively, of the strong cold-resistant cultivar GC0209D. Root-2, Stem-2, and Leaf-2 represent the roots, stems, and leaves, respectively, of the cold-sensitive cultivar GC0265D.

This study evaluated the patterns of expression of 13 genes in different tissues of the two cultivars and found that the expression patterns of 11 genes were the same in the two cultivars. The expression patterns of *PmJAZ*, *PmAP22*, *PmAP23*, *PmWRKY1*, *PmWRKY2*, *PmPRX5*, and *PmPRX4.1* were similar in different tissues; these genes showed upregulated expression in stems and downregulated expression in leaves compared to roots. The expression levels of *PmBAM1*, *PmBAM2*, and *PmWRKY22* in stems and leaves were higher than those in roots. The expression patterns of *PmPRX4.1* and *PmEIN3* differed somewhat in the two cultivars. *PmPRX4.1* was upregulated in the stems and downregulated in the leaves of GC0209D and upregulated in both the stems and leaves of GC0265D, while *PmEIN3* was upregulated in the stems and leaves of GC0209D, upregulated in the stems of GC0265D, and downregulated in the leaves of GC0265D (**Figure 4**).

### Starch and sucrose metabolic pathways and cold resistance in *P. massoniana*

**Figure 5:**
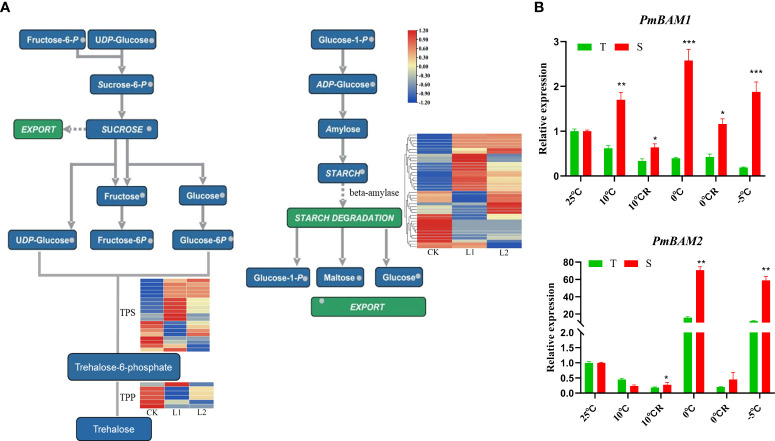
Relationship of starch and sucrose metabolism pathways with cold resistance in *P. massoniana*. **(A)** Enrichment of differentially expressed genes in starch and sucrose metabolic pathways. **(B)** Expression of *PmBAMs* in roots of *P. massoniana* differing in cold resistance. *P < 0.05, Student t tests, **P < 0.01, Student t tests, ***P < 0.001, Student t tests.

With decrease in temperature, starch grains accumulated continuously in the roots of the plants. However, in the plants exposed to a temperature of -5°C, the root tip cells collapsed and lysed, and large amounts of cell contents exuded. Therefore, no starch grains were found in the root tip cells of those plants. In the plants in the 0°C R group, the number of starch grains was higher, and in the GC0209D cultivar, starch grain number was significantly higher than that in the control (**Figure 2**). The starch and sucrose pathways involve the genes that encode glucan endo-1,3-beta-glucosidase (β-1,3-glucosidase) and beta-amylase (BAM). Of the 41 β-1,3-glucosidase genes, 27 were upregulated and 14 were downregulated, and the 39 BAM genes were all upregulated (**Figure 5A**, **Supplementary Figure 4**). Expression analysis of the *PmBAM1* and *PmBAM2* genes by qRT−PCR showed that the expression of both genes was induced by exposure of the plants to low temperature and that their expression patterns differed. *PmBAM1* showed opposite expression patterns in the two *P. massoniana* cultivars, whereas the pattern of *PmBAM2* expression was the same in the two cultivars, i.e., its level of expression first increased and then decreased with decreasing temperature; however, its level of expression was significantly higher in GC0265D than in GC0209D (**Figure 5B**, **Supplementary Table 4**).

### The phenylpropanoid metabolic pathway and cold resistance in *P. massoniana*

**Figure 6:**
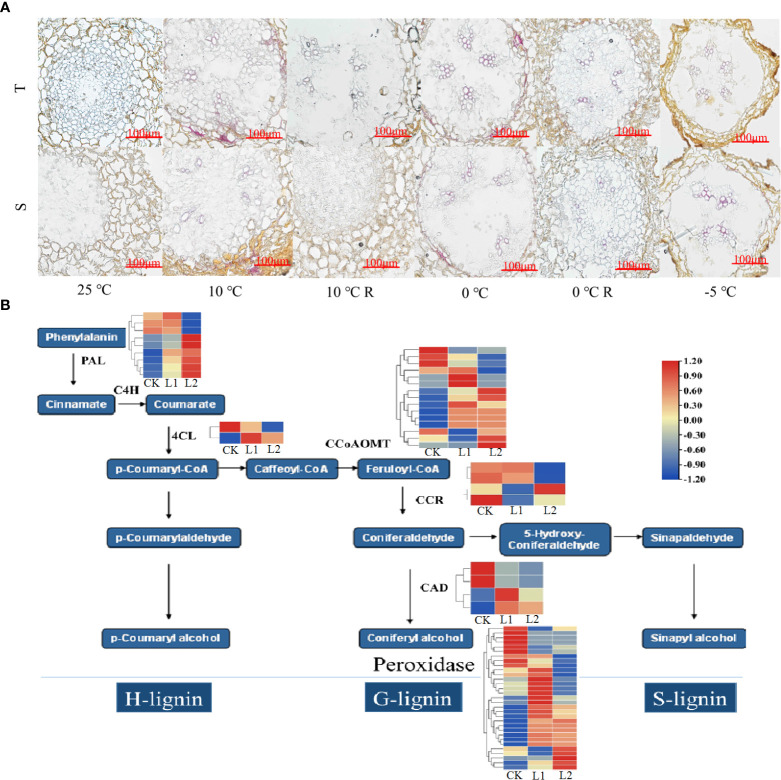
Relationship between the phenylpropane metabolic pathway and cold resistance of *P. massoniana*. **(A)** Effects of low-temperature stress on lignin content in roots of *P. massoniana* seedlings. En, endodermis; Xy, xylem. **(B)** Enrichment of differentially expressed genes in the lignin synthesis pathway.

No lignin staining was observed in cross-sections of the roots of the control plants (**Figure 6A**), but lignin gradually accumulated in the endothelial cell walls and xylem of the roots as the temperature decreased. In GC0209D plants subjected to 0°C stress, staining of the endothelial cell walls and xylem of the roots by phloroglucinol solution was significantly more intense than that observed in the control or in the GC0265D cultivar after 0°C stress, indicating that the lignin content of the roots of the highly cold-resistant cultivar increased significantly. When the temperature reached -5°C, the endothelial cells collapsed and lysed; therefore, no lignin was detected in the endothelial cell walls, but lignin was detected in the xylem. Genes in the phenylpropanoid metabolic pathway that were significantly upregulated under low-temperature stress were identified as *POD*, shikimate O-hydroxycinnamoyl transferase (*HCT*), 4-coumarate: CoA ligase (*4CL*), caffeoyl-CoA 3-O-methyltransferase (*CCoAOMT*), cinnamoyl-CoA reductase (*CCR*), phenylalanine ammonia-lyase (*PAL*), and cinnamyl alcohol dehydrogenase (*CAD*) (**Figure 6B**, **Supplementary Figure 5**), which are genes that play extremely important roles in the lignin biosynthesis pathway. Thus, the results indicate that the lignin biosynthesis pathway plays an important role in the response of *P. massoniana* roots to low-temperature stress. In addition, the expression of *PmPRX5* was downregulated in GC0209D and upregulated significantly in GC0265D in plants exposed to low-temperature stress below 0°C, and during the recovery period, *PmPRX5* expression was not upregulated in either of the two cultivars (**Supplementary Figure 6**). The expression levels of the three *PmPRX* genes were significantly higher in GC0265D than in GC0209D under various temperatures, further indicating that there are specific regulatory mechanisms in different *P. massoniana* cultivars.

### TFs and cold resistance in *P. massoniana*

**Figure 7:**
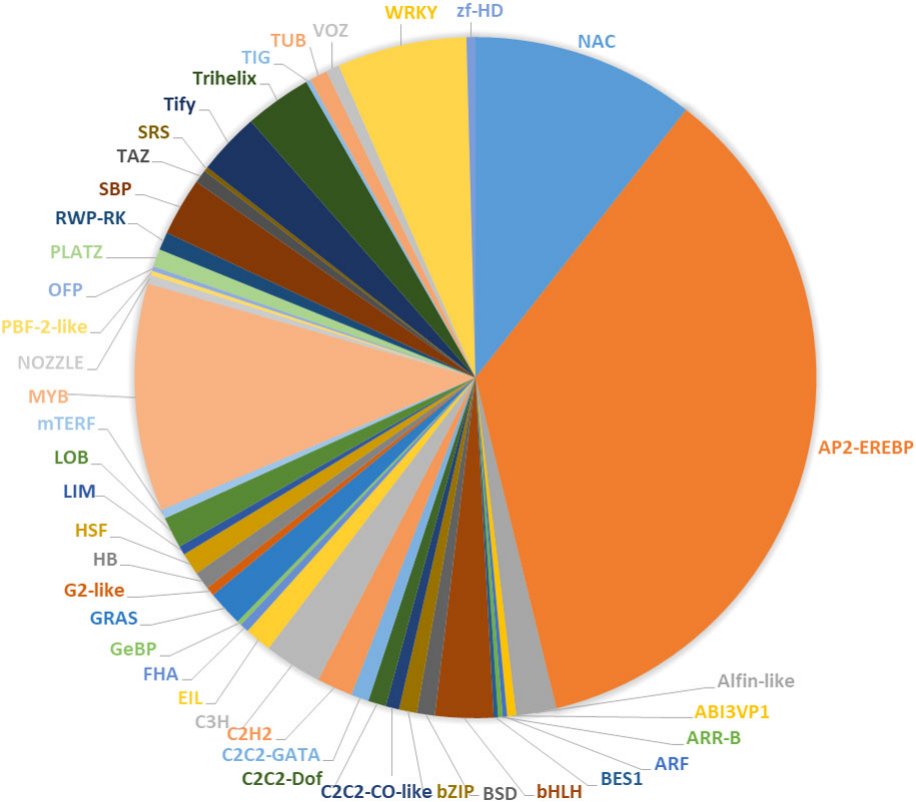
Relationship between transcription factors and cold resistance in *P. massoniana*.

**Figure 8:**
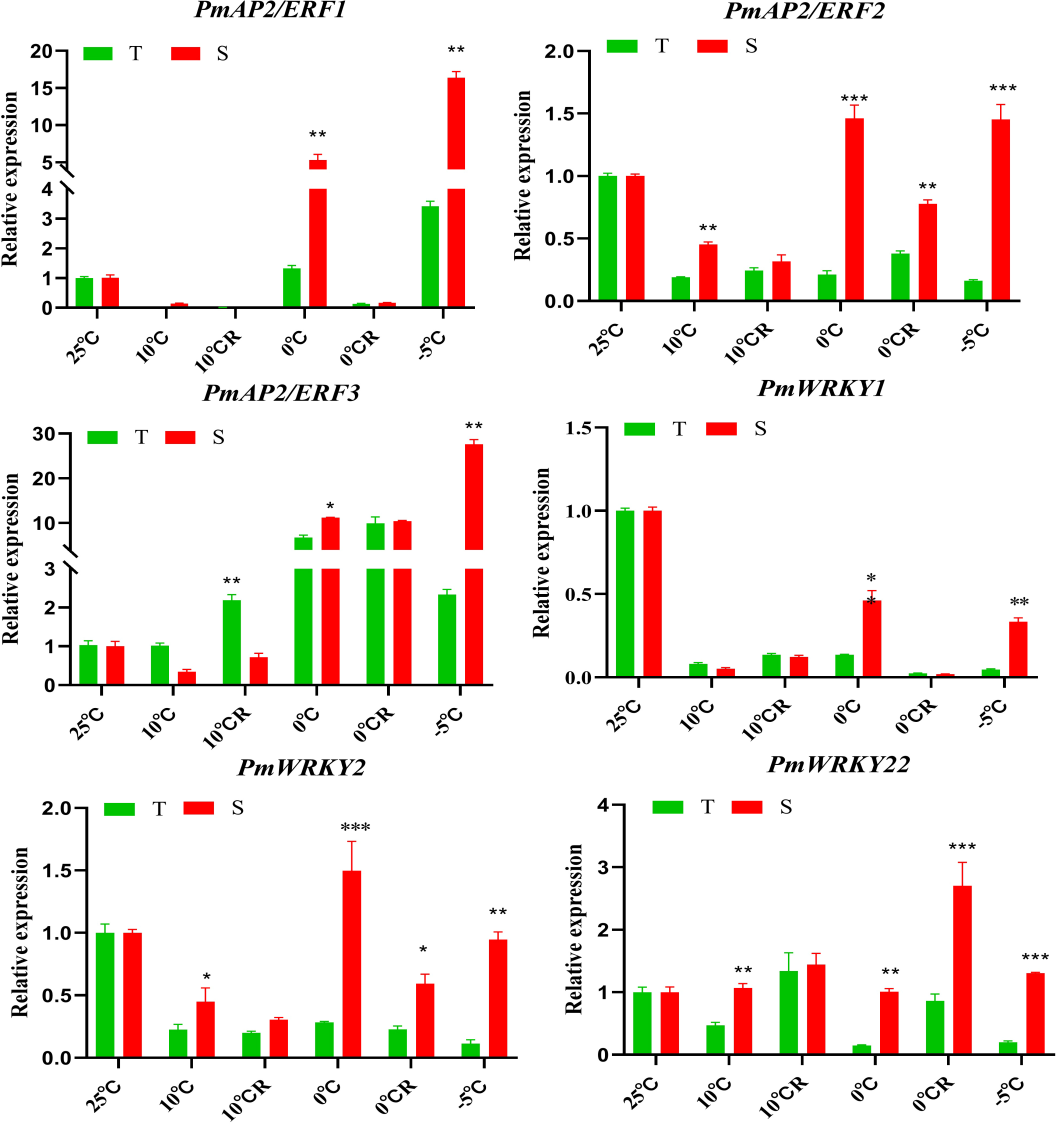
Expression of *PmAP2s* and *PmWRKYs* in roots of *P. massoniana* differing in cold resistance. *P < 0.05, Student t tests, **P < 0.01, Student t tests, ***P < 0.001, Student t tests.

The results of this study indicate that 390 TFs may be involved in low-temperature stress; 233 of these were upregulated, and 157 were downregulated. The TFs whose expression was affected by low-temperature stress were distributed among 41 TF families (**Figure 7**). The largest number of these TFs belonged to the AP2/ERF gene family (168 DEGs), followed by the MYB family (51 DEGs), the NAC family (50 DEGs), and the WRKY family (29 DEGs) (**Figure 7**, **Supplementary Figures 1**, **7**). bHLH, TIFY, trihelix, C3H, and Alfin-like TFs were also induced by low-temperature stress (**Figure 7**). We found highly expressed genes in the AP2/ERF, WRKY, and NAC family (**Supplementary Figures 1**, **7**). For example, the isoform_33369, isoform_19668, isoform_22683 and isoform_45231 of AP2/ERF family, the isoform_40963, isoform_270370, isoform_6954 and isoform_27027 of WRKY family, and the isoform_23455, isoform_99345, isoform_13354 of NAC family, respectively. Although, MYB transcription factors under was not found genes of coincidence FDR ≤ 0.001 and |log2FC|≥2 in low temperature stress, but We found some significant genes in the MYB, For example, isoform_275883, isoform_66794, and isoform212192 (**Supplementary Figure 7**). Under low-temperature stress conditions, the expression of *PmAP21* and *PmAP23* increased with decreasing temperature and differed in the two *P. massoniana* cultivars (**Figure 8**); *PmAP22* expression was suppressed in GC0209D and upregulated in GC0265D, and *PmAP23* expression was maintained at a high level in GC0209D compared to the control. *PmWRKY1* expression was upregulated in both cultivars under low-temperature stress conditions (**Figure 8**), while *PmWRKY2* showed a pattern consistent with that of *PmWRKY1* in GC0209D. However, *PmWRKY2* expression was only significantly upregulated at 0°C in GC0265D; it was downregulated during low-temperature stress in GC0209D and was not significantly different from that in the control in GC0265D (**Figure 8**). These results indicate that different pathways of resistance to low-temperature stress exist in different *P. massoniana* cultivars (**Figure 8**, **Supplementary Table 4**).

### Hormone signal transduction pathways and cold resistance in *P. massoniana*

**Figure 9:**
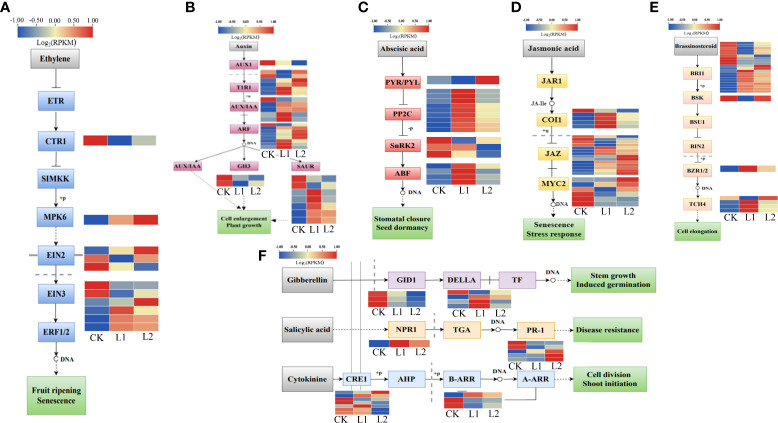
Relationship between hormone metabolism pathways and cold resistance in *P. massoniana*. **(A–F)** Enrichment of differentially expressed genes in plant hormone signal transduction pathways.

A total of 141 DEGs associated with plant hormone signal transduction pathways were enriched in plants subjected to low-temperature stress. The genes related to endothelin (ET) signal transduction included one downregulated gene that encodes the protein kinase copper transporter 1 (CTR1), one upregulated gene that encodes the protein kinase MAPK 6, and six ETHYLENE INSENSITIVE3 (EIN3) and three ETHYLENE INSENSITIVE2 (EIN2) TFs (**Figure 9A**). The expression of 29 indoleacetic acid (IAA)-responsive genes changed significantly under low-temperature stress, and the overall expression levels of the [TRANSPORT INHIBITOR RESPONSE 1 (*TIR1*) auxin receptor, auxin response factors (*ARFs*), and the auxin-responsive genes *Aux/IAA* and *SAURs* were upregulated (**Figure 9B**). Fifteen genes were annotated to the abscisic acid (ABA) signal transduction pathway; of these, four genes encode ABF TFs that recognize ABA response elements. Nine ABF TFs that affect the expression of protein phosphatase 2C (*PP2C*) were upregulated. Three ABF TFs that affect the expression of sucrose nonfermenting 1-related protein kinase 2 (*SnRK2*) were downregulated, and one of the annotated genes belongs to the pyrabactin resistance-like (PYL) family of ABA receptors that allow plants to respond to low-temperature stress through the AREB/ABF-SnRK2 pathway (**Figure 9C**). Among the 15 jasmonic acid (JA)-related genes, 14 were JASMONATE ZIM-DOMAIN (*JAZ*) genes, and 11 of them were upregulated (**Figure 9D**). Twenty-six genes in the brassinosteroid (BR) signal transduction pathway were also involved in low-temperature responses; of these, brassinosteroid insensitive 1 (*BRI1*) was both positively and negatively regulated, and *TCH4s* were upregulated (**Figure 9E**). This study also detected seven DEGs related to the gibberellic acid (GA) signaling pathway, six genes related to the salicylic acid (SA) signaling pathway, and 10 genes related to the cytokinin (CTK) signaling pathway (**Figure 9F**). Different hormone signal transduction pathways participate in the defense against cold stress by activating gene expression. The expression patterns showed that the expression of *PmEIN3* and *PmJAZ* in the two *P. massoniana* cultivars decreased with decreasing temperature and that these genes were upregulated when the temperature increased; the expression levels of *PmEIN3* and *PmJAZ* were lower in GC0209D than in GC0265D (**Supplementary Figure 6**, **Supplementary Table 4**).

## Discussion

### Changes in the physiological and biochemical indexes of *P. massoniana* seedlings in response to low-temperature stress

When plants adapt to low-temperature environments, a series of physiological changes occur. These include a decrease in the selective permeability of cell membranes, an increase in the relative conductivity, and a weakening of the ability of leaves to undergo stomatal closure, resulting in severe dehydration of the cells (Bloom et al., 2004; Karami-Moalem et al., 2018; Zhang et al., 2019). To maintain the water balance of cells, plants actively accumulate substances such as proline, soluble sugars, and soluble proteins. Increasing the concentration of intracellular solutes in this manner reduces the water potential and improves the water absorption capacity of cells (Kumar and Yadav, 2008; Hare et al., 2010; Nejadsadeghi et al., 2014; Zhang et al., 2018; Airaki et al., 2012; Zheng et al., 2015; Zhao et al., 2017; Peng et al., 2019). In this study, the proline, soluble sugar, and soluble protein contents of the two strains increased overall when the plants suffered from low-temperature stress, indicating that under low-temperature stress, *P. massoniana* seedlings increase the amounts of osmoregulatory substances in their roots to resist low-temperature stress.

Low temperature can also lead to the production of excessive ROS. Plants activate their protective enzyme systems to increase the activity of antioxidant enzymes such as SOD, POD, and catalase (CAT), which remove excess ROS (Jahnke et al., 2006; Xu et al., 2014; Hu et al., 2016; Li et al., 2018; Nasef, 2018; Kaur et al., 2018; Peng et al., 2019). However, if low-temperature conditions continue to intensify, many harmful membrane-lipid-peroxidation products, such as MDA, accumulate (Zhang et al., 2013; Chen et al., 2015). In this study, the SOD and POD activities and MDA contents of the two strains increased to resist low-temperature stress. However, the SOD and POD activities at -5°C treatment were significantly lower than those of the control, indicating that SOD and POD could no longer scavenge ROS to prevent damage to cell membranes in a timely manner (Santini et al., 2013; Xu et al., 2014; Hu et al., 2016; Kaur et al., 2018). In GC0209D, the proline content, soluble sugar content, and POD activity under low-temperature stress were significantly higher than those in the cold-sensitive cultivar GC0265D, while the MDA content was significantly lower than that in GC0265D, indicating strong cold resistance (Kumar and Yadav, 2008; Santini et al., 2013; Nejadsadeghi et al., 2014).

After the recovery period, the soluble sugar and protein contents and the POD and SOD activities of the two cultivars increased, and the MDA content was significantly lower than that in plants that had only received the 0°C treatment. This finding indicates that after appropriate low-temperature treatment, the plants were able to strengthen their defenses by retaining stress memory to increase their cold resistance, a process that is similar to cold hardening (Li et al., 2002; Avramova, 2015; Andrew et al., 2018).

### Responses of related metabolic pathway networks and genes to cold stress in *P. massoniana*

In a low-temperature environment, plants regulate the production of specific proteins at the transcriptional level to guide the production of metabolites by regulating gene expression, thereby responding to stress (Lee and Young, 2000; Agarwal and Jha, 2010). The DEGs detected in this study were enriched mainly in the phenylpropanoid biosynthesis pathway, the starch and sucrose metabolic pathways, and the plant hormone synthesis pathway.

### Responses of the starch and sucrose metabolic pathways of *P. massoniana* to cold stress

When plants are exposed to low-temperature stress, they accumulate nutrients and metabolites that improve their cold resistance. Among these nutrients and metabolites, stored sugars are present in plants in the form of starch granules. When stress intensifies, starch granules are hydrolyzed into soluble sugars, which increases the intracellular sugar content and thereby improves the cold resistance of plant cells (Zheng et al., 2015; Peng et al., 2019). This study found that the content of starch grains in the GC0209D cultivar increased when the plants were exposed to cold stresses of 10°C and 0°C, indicating that low temperatures promoted the synthesis of starch grains in plants. When the temperature reached -5°C, very few starch grains were observed, and it is possible that sustained low-temperature stress caused rapid hydrolysis of starch grains into soluble sugars to enhance cold resistance.

Studies have shown that expression of *BAM* genes improves the cold resistance of plants and increases their soluble sugar content (Kaplan and Guy, 2005; Peng et al., 2014) and can also play a role in scavenging ROS and maintaining cell homeostasis (Couée et al., 2006; Zhao et al., 2019). This study found that the expression of the *PmBAM* gene was upregulated under low-temperature stress conditions, and further GO analysis showed that its expression was related to amylase activity (GO:0016160). Based on the trends in soluble sugar content, it is speculated that in plants exposed to low-temperature stress, β-amylase activity can be increased through regulation of the expression of *BAM* genes in this manner. Soluble sugar content can increase to improve cold resistance (Xin and Browse, 2000; Valerio et al., 2011; Zanella et al., 2016; Liu et al., 2022). This study found 19 *PmTPS* and six *PmTPP* genes among the DEGs. It has been confirmed that the *OsTPS1* and *OsTPP1* genes enhance cold resistance in rice (Chary et al., 2008); however, whether they play roles in the response of *P. massoniana* to low-temperature stress requires further study.

### Responses of the phenylpropanoid biosynthesis pathway to cold stress in *P. massoniana*

The phenylpropanoid biosynthesis pathway plays an important role in plants exposed to low-temperature stress (Pu et al., 2019). Many of the DEGs identified in this study were enriched in the phenylpropanoid biosynthesis pathway. Lignin is one of the final products of the phenylpropanoid metabolic pathway and plays an important role in improving plant resistance (Liu et al., 2018; Liang et al., 2022). POD is a key enzyme in lignin biosynthesis (Barros et al., 2019). This study found that the genes involved in POD activity were most numerous among all of the DEGs detected in this study. Their expression levels were also the highest among these DEGs. Previous studies have shown that the expression levels of *PAL*, *C4H*, *4CL1*, *4CL2*, *CAD1*, *HCT3*, *CCoAOMT*, *POD1*, and *POD2* were all high during periods when lignin biosynthesis was high (Luo and Li, 2022). In this study, the overall expression levels of *PmPAL, Pm4CL, PmCAD, PmHCT*, *PmCCoAOMT*, and *PmPOD* were upregulated in plants exposed to low-temperature stress, and the lignin content was relatively high, indicating that *PmPRXs* may be involved in lignin biosynthesis during low-temperature stress. In combination with the results regarding lignin content, this observation further shows that it is very possible to enhance the cold resistance of plants exposed to low-temperature stress by regulating the expression of genes in the lignin biosynthesis pathway.

### Responses of TFs to cold stress in *P. massoniana*

It is indisputable that TFs regulate plant responses to stress and improve their ability to adapt to stress (Agarwal et al., 2010; Guo et al., 2018; Vergara et al., 2022). The *CBF* gene, a member of the AP2/EREBP family that acts in the CBF/DREB1-dependent cold signaling pathway in plants, initiates the response system of plants to cold stress by regulating the expression of the downstream cold-responsive (*COR*) gene (Shinozaki, 2000; Hu et al., 2013; Jia et al., 2016; Lv et al., 2019). NAC transcription factors play important roles in plant abiotic stress, with most genes regulating stress response (Hu et al., 2008; Lei et al., 2016; Lei et al., 2016; Zhuo et al., 2018), but a small number of NAC genes negatively regulate in low-temperature stress (An et al., 2018). WRKY TFs also play an important role in the resistance of plants to adverse environments (Rushton et al., 2012; Wang et al., 2014; Zhang et al., 2016). The *MYB* gene enhances plant resistance by regulating the expression levels of stress-response genes (Zhu et al., 2005; Liao et al., 2008; Wang et al., 2019). In this study, the expression of three *PmAP2* genes, three *PmWRKY* genes, and three *PmNAC* genes was induced significantly under low-temperature stress conditions, indicating that these genes are involved in the response of *P. massoniana* to low-temperature stress. The precise functions of these genes should be investigated in future studies.

### Responses of plant hormone signal transduction pathways to cold stress in *P. massoniana*

When plants are under stress, plant hormones act as endogenous signal transduction molecules, transmitting stress signals to multiple hormone response pathways and permitting rapid adjustment of the expression of related genes and of the contents of intracellular substances to resist the damage caused by stress (Chinnusamy et al., 2004; Pociecha et al., 2008). This study found that the genes associated with the GA and JA pathways were downregulated, while the genes associated with the IAA, ABA, BR, and ET pathways were upregulated. These results indicate that plants respond to low-temperature stress by regulating the levels of expression of these signal transduction pathway-related genes. Overexpression of *Arabidopsis EIN3* reduces cold resistance (Liu et al., 2018). In this study, the expression of *PmEIN3* was upregulated in the two cultivars when the plants were exposed to low-temperature stress, and it is speculated that downregulation of *PmEIN3* expression may improve the plants’ cold resistance. Low-temperature stress can activate the transcriptional activity of DREB1/CBF TFs through activation of *JAZ* expression in the JA signaling pathway, thereby regulating the expression of the downstream gene *COR* and improving the cold resistance of plants (Hu et al., 2013). In this study, the pattern of *PmJAZ* expression differed from that in other *P. massoniana* cultivars, and there may be additional regulatory patterns that will require further study through transcriptome sequencing of various *P. massoniana* cultivars.

## Conclusion

This study reveals new insights on the regulation of responses to low-temperature stress in *P. massoniana*. These data highlight the effects of low-temperature stress on phenylpropanoid biosynthesis pathways, starch and sucrose metabolism pathways, and plant hormone signal transduction pathways, as well as transcription factor-related genes. These are critical for *P. massoniana* responses to low-temperature stress. In particular, it was found that peroxidase regulated lignin synthesis and PmBAMs regulated starch and sucrose synthesis in response to cold stress in *P. massoniana*. The genes encoding these enzymes could be potential targets for biotechnological strategies aimed at providing new varieties resistant to low temperature.

## Data availability statement

The datasets presented in this study can be found in online repositories. The names of the repository/repositories and accession number(s) can be found below: NCBI BioProject accession number: PRJNA881065.

## Author contributions

JL, HC, and ZY designed the study. SS, QL, JX, and JT performed the analyses. JL, HC, and ZY wrote the manuscript. All authors contributed to the article and approved the submitted version.

## Funding

HC received funding from the Natural Science Foundation of China (32060348, 31660219), Bagui Young Scholars Program, and the Guangxi Natural Science Foundation (Grant 2018GXNSFAA294057, 2019GXNSFDA245033). ZY received funding from Bagui Scholars Program (2019A26) and the Guangxi Science and Technology and Talents Special Project (AD19254004).

## Conflict of interest

The authors declare that the research was conducted in the absence of any commercial or financial relationships that could be construed as a potential conflict of interest.

## Publisher’s note

All claims expressed in this article are solely those of the authors and do not necessarily represent those of their affiliated organizations, or those of the publisher, the editors and the reviewers. Any product that may be evaluated in this article, or claim that may be made by its manufacturer, is not guaranteed or endorsed by the publisher.

## Supplementary material

Supplementary Figure 1 AP2/ERF and WRKY Transcription Factor genes expression in roots of *P. massoniana* in cold resistance. **(A)** AP2/ERF Transcription Factor, isoform_33369, isoform_19668 and isoform_45231 were for *PmAP2/ERF1*, *PmAP2/ERF3* and *PmAP2/ERF2*, respectively. **(B)** WRKY ranscription Factor, isoform_40963, isoform_6954 and isoform_27027 were for *PmWRKY1*, *PmWRKY2* and *PmWRKY22*.

Supplementary Figure 2 Thermal correlation diagram for each sample.

Supplementary Figure 3 GO enrichment analysis of differentially expressed genes.

Supplementary Figure 4 Heatmap of differentially expressed genes in starch and sucrose metabolic pathways.

Supplementary Figure 5 Heatmap of differentially expressed genes in phenylpropanoid biosynthesis pathways.

Supplementary Figure 6 Expression of *PmPRXs*, *PmEIN3*, and *PmJAZ* in roots of *P. massoniana* differing in cold resistance. * *P* < 0.05, Student t tests, ** *P* < 0.01, Student t tests, *** *P* < 0.001, Student t tests.

Supplementary Figure 7 NAC and MYB Transcription Factor genes expression in roots of *P. massoniana* in cold resistance. **(A)** NAC Transcription Factor. **(B)** MYB Transcription Factor.
