# Supplementary figures and images for: Physiological and molecular mechanisms of the response of roots of Pinus massoniana Lamb. to low-temperature stress
Source: Front Plant Sci. 2022 Sep 28;13:954324. doi: 10.3389/fpls.2022.954324 (PMC9554314; doi:10.3389/fpls.2022.954324)

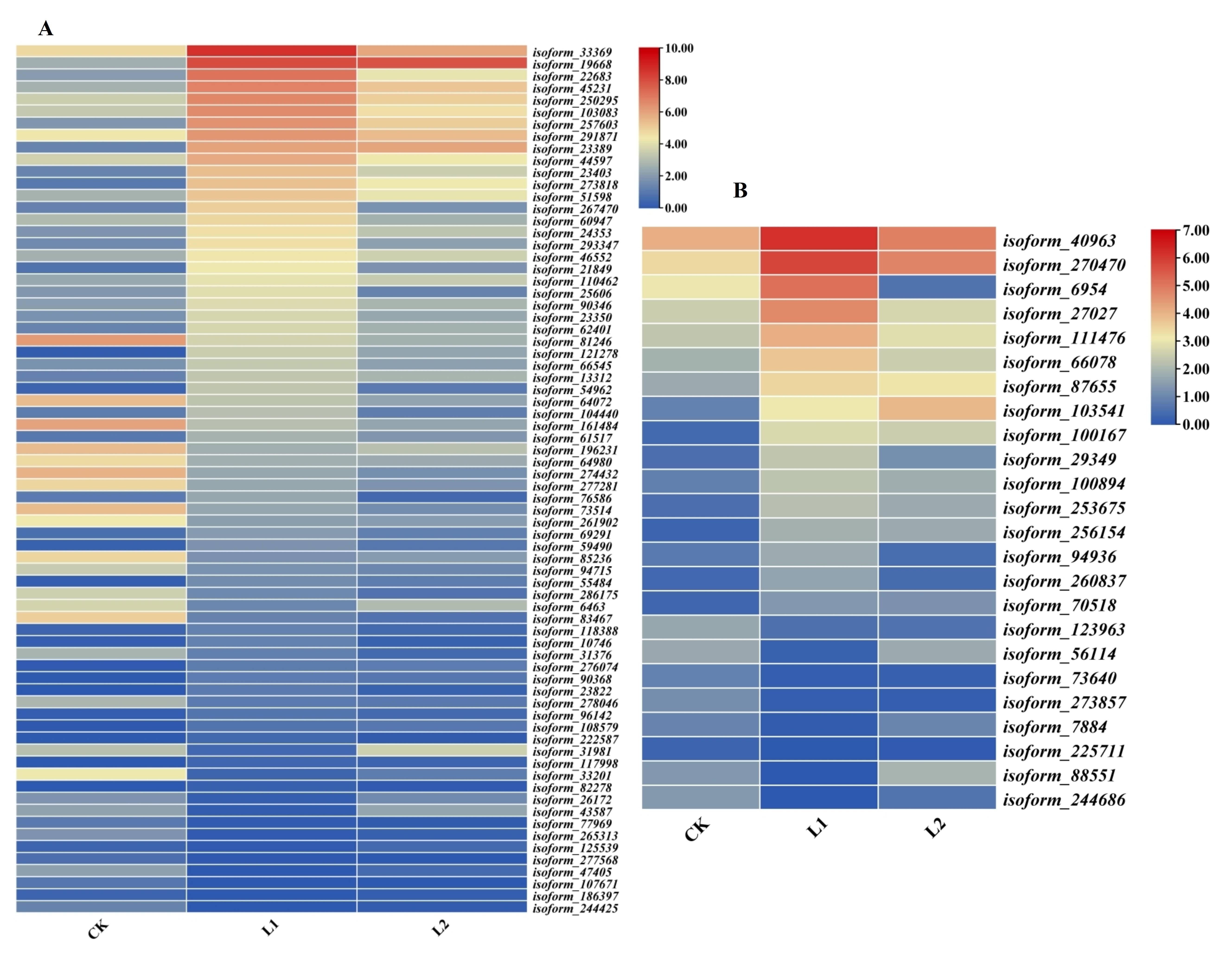

Supplement: Supplementary Figure 1 — AP2/ERF and WRKY Transcription Factor genes expression in roots of P. massoniana in cold resistance. (A) AP2/ERF Transcription Factor, isoform_33369, isoform_19668 and isoform_45231 were for PmAP2/ERF1, PmAP2/ERF3 and PmAP2/ERF2, respectively. (B) WRKY ranscription Factor, isoform_40963, isoform_6954 and isoform_27027 were for PmWRKY1, PmWRKY2 and PmWRKY22. [file Image_1.png]

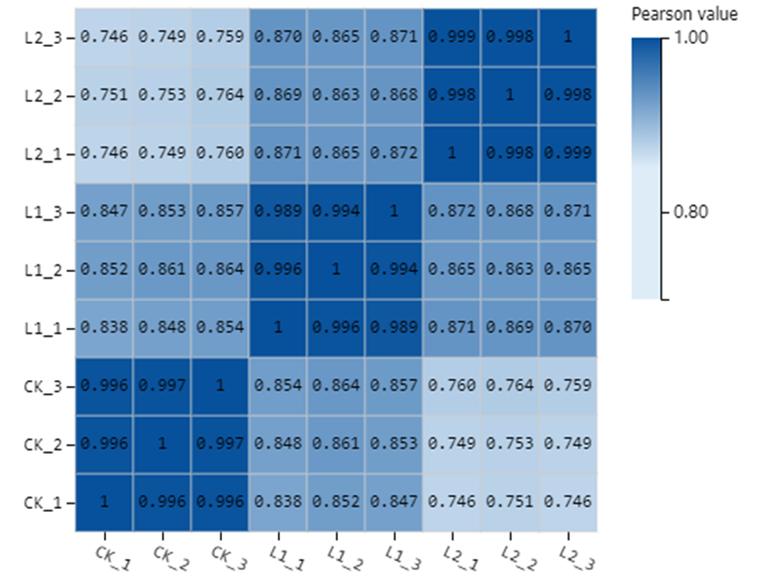

Supplement: Supplementary Figure 2 — Thermal correlation diagram for each sample. [file Image_2.jpg]

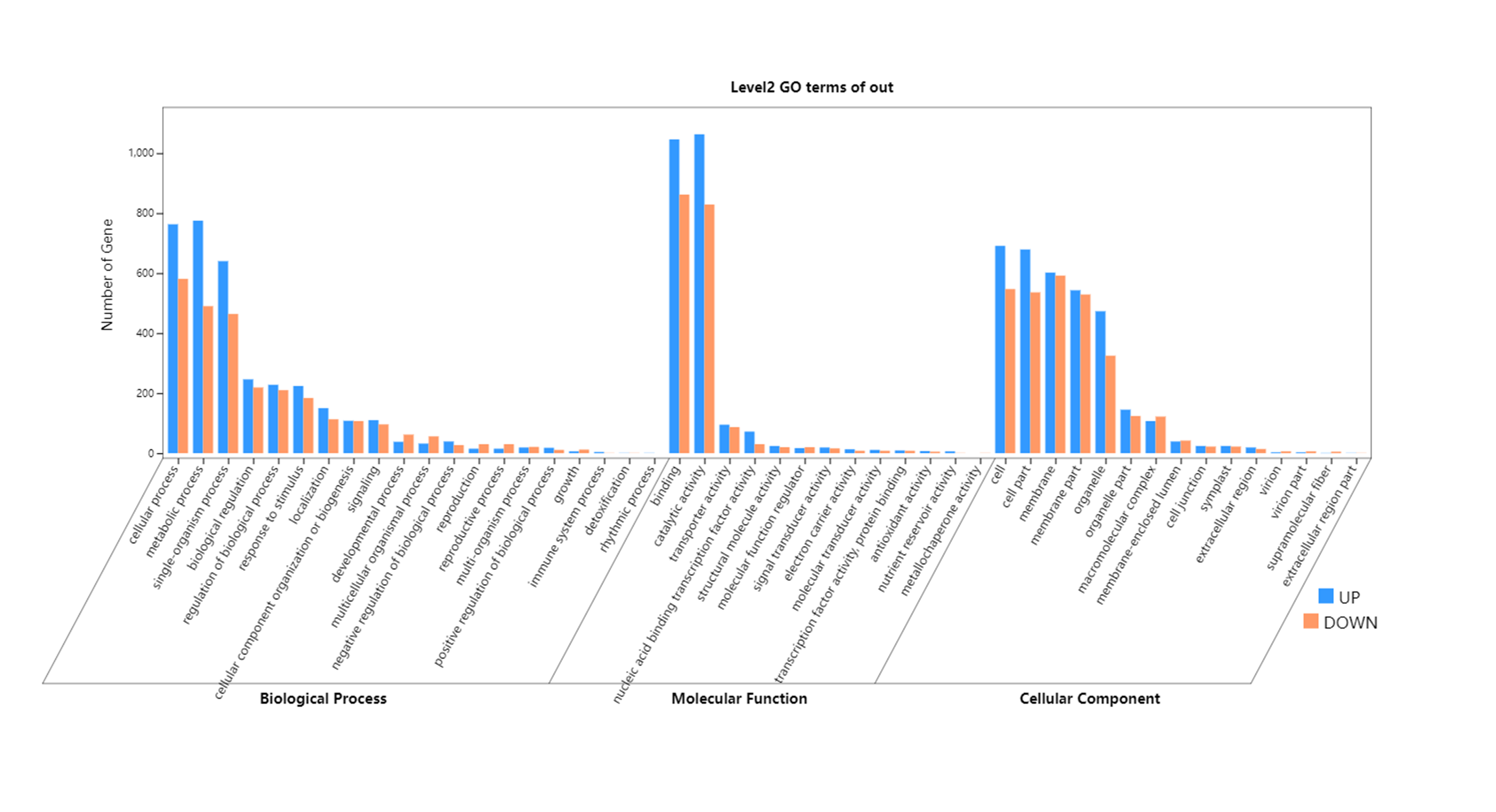

Supplement: Supplementary Figure 3 — GO enrichment analysis of differentially expressed genes. [file Image_3.tif]

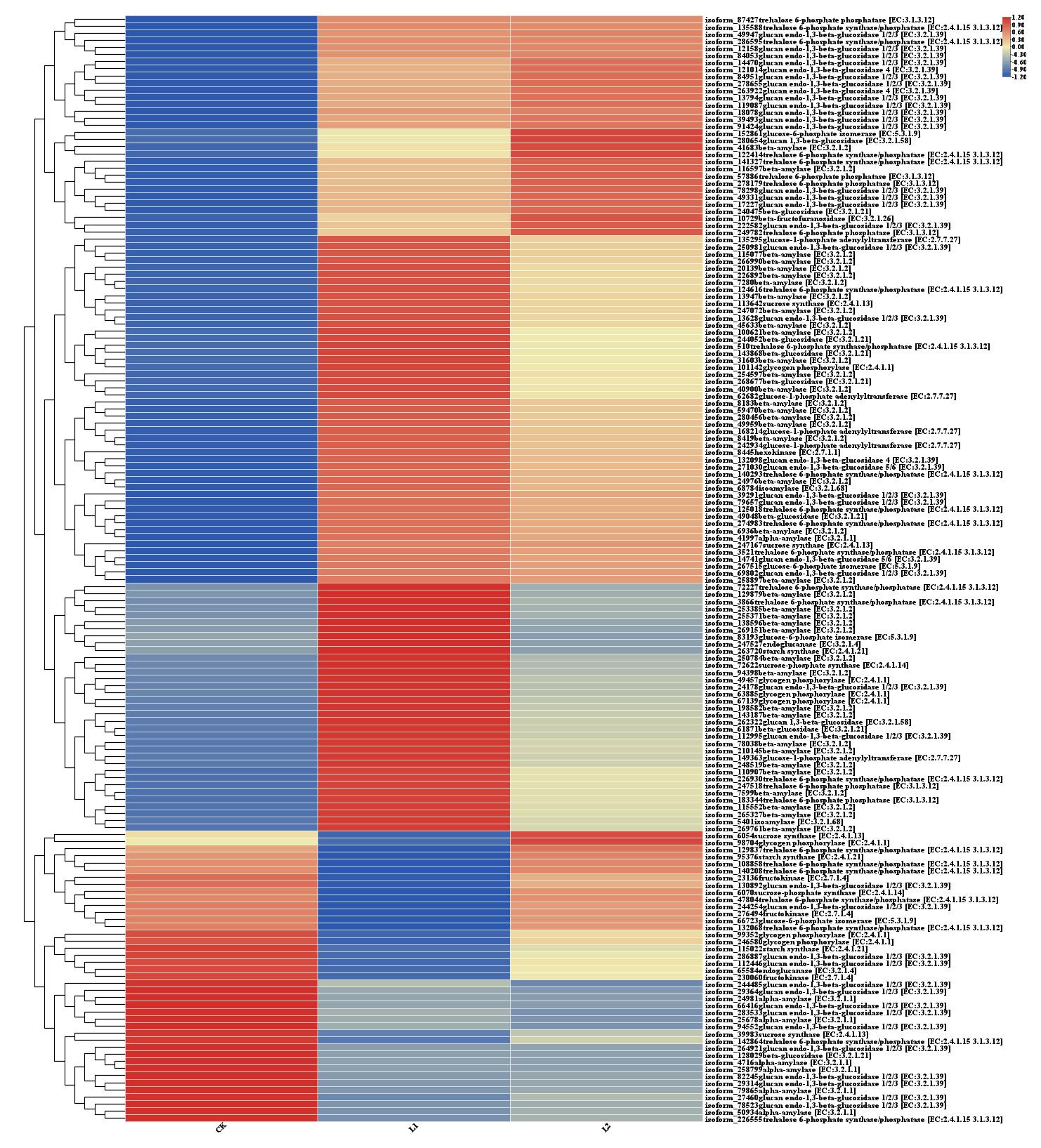

Supplement: Supplementary Figure 4 — Heatmap of differentially expressed genes in starch and sucrose metabolic pathways. [file Image_4.jpg]

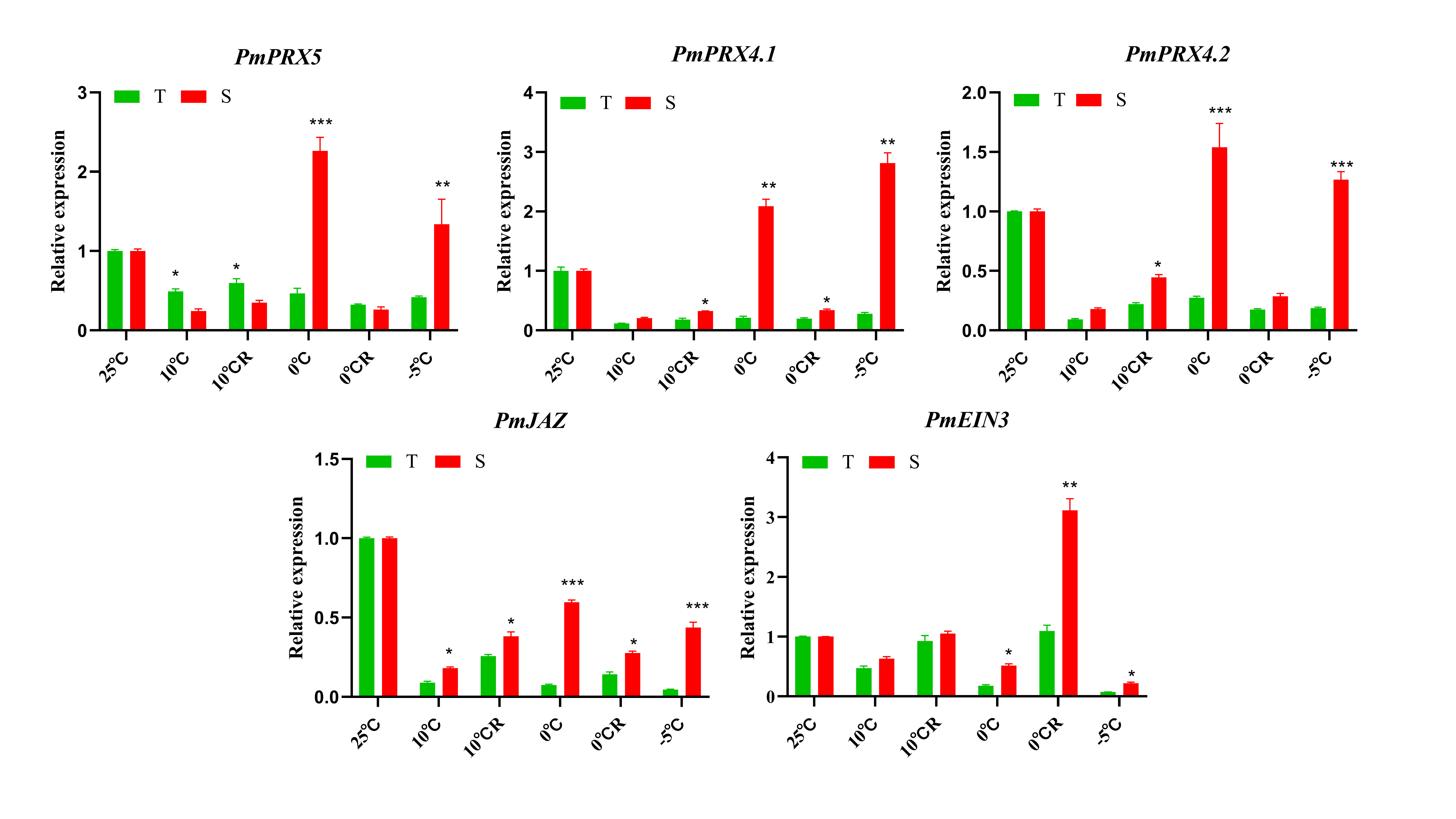

Supplement: Supplementary Figure 6 — Expression of PmPRXs, PmEIN3, and PmJAZ in roots of P. massoniana differing in cold resistance. * P < 0.05, Student t tests, ** P < 0.01, Student t tests, *** P < 0.001, Student t tests. [file Image_6.tif]

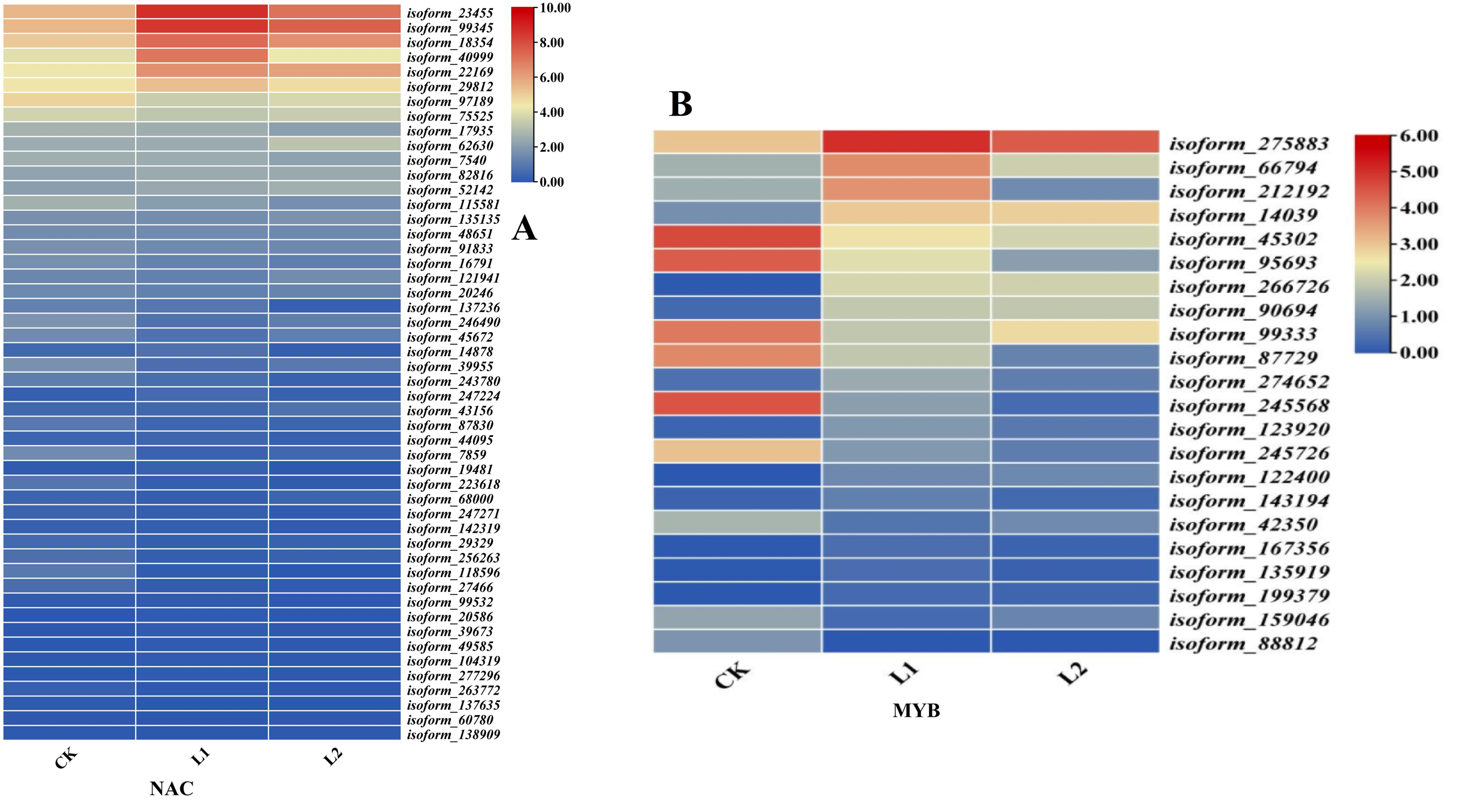

Supplement: Supplementary Figure 7 — NAC and MYB Transcription Factor genes expression in roots of P. massoniana in cold resistance. (A) NAC Transcription Factor. (B) MYB Transcription Factor. [file Image_7.jpg]
