# Supplementary material for: Physiological and molecular mechanisms of the response of roots of Pinus massoniana Lamb. to low-temperature stress
Source: Front Plant Sci. 2022 Sep 28;13:954324. doi: 10.3389/fpls.2022.954324 (PMC9554314; doi:10.3389/fpls.2022.954324)
Supplement: Supplementary file 8 [file Table_1.doc]

**Supplementary Table 2 qRT-PCR primer sequences**

| Genes | Transcripts ID | Forward prime | Reverse prime |
| --- | --- | --- | --- |
| *Peroxidase* (*PRX5*) | isoform_87369 | CAAGTCACCACCTACAGCTCCAATC | GGATTTGTCCGCTCGTACCAGTG |
| *Peroxidase* (*PRX4.1*) | isoform_29056 | CCATTGCTGCTCGTGACTCTATCG | GCGGGAGATGGAATGTTGTTGTTTG |
| *Peroxidase* (*PRX4.2*) | isoform_253777 | TAGTGAAAGCCGCAGTGACCAAAG | CATCGCAGCCATTAACGAAGCAATC |
| *AP2/ERF1* | isoform_19668 | GCCAGCGGGTTCATCATCAGAAG | TCTGCCTTACGCCTCTGTACCG |
| *AP2/ERF2* | isoform_33369 | GCAACAAACCGACATGGCTAAACC | TTCGTATTTCCGACACCCATTTCCC |
| *AP2/ERF3* | isoform_45231 | TCAGGAAACCCTAGCCCTTCGG | CTCTTGCTCCATTGTCGCCTCTTC |
| *WRKY1* | isoform_4096 | GAGAATCGTAGCAGAGCGGACAAG | CGTCATCTTTTGCCCATACTTTCGC |
| *WRKY2* | isoform_6954 | CTGAAGGCTGTGGTGCTCTTGTC | TTGATTGCTCCGGTGTACCTTGTG |
| *WRKY22* | isoform_27027 | TCTCTCAGCCCAACAGCCGTAG | CAGCGTCCGCCTGTAGTGTTAC |
| *beta-amylase*(*BAM1*) | isoform_266990 | ATTGTGATGCTGCCTCTGGATACTG | CCCACCACACGTCCACCATTAC |
| *beta-amylase*(*BAM2*) | isoform_24976 | GAGTGCTGGTGTTGAGGGAGTTATG | CAATCCGTGTTTCTTCGCCATTTCC |
| *EIN3* | isoform_6518 | TTGAGAAGCAGATGTGGAAGGATCG | TTCGGGCTTGTTCTTGTGACTGTC |
| *JAZ* | isoform_28402 | CAGCGAAAGCTACGGAGCAACC | GCTGGCACATCGTAAACATTCACC |
| *PmUBI4* |  | AGCTCCGACACCATTGATAA | CCAAAGTACGTCCATCTTCCA |
| *PmCYP* |  | CAAGGGTTCGTCGTTCCAC | GGCAAACTTCTCGCCGTA |
