# Supplementary material for: Physiological and molecular mechanisms of the response of roots of Pinus massoniana Lamb. to low-temperature stress
Source: Front Plant Sci. 2022 Sep 28;13:954324. doi: 10.3389/fpls.2022.954324 (PMC9554314; doi:10.3389/fpls.2022.954324)
Supplement: Supplementary file 10 [file Table_3.doc]

**Supplementary Table 1 Transcriptome sequencing quality and comparison reference genome statistics**

| sample | raw reads | clean reads | Q20(%) | Q30(%) | GC Content(%) | Mapped Reads(%) | |
| --- | --- | --- | --- | --- | --- | --- | --- |
| CK_1 | 63,239,772 | 55,879,562 | 96.63 | 88.3 | 45.4 | | 75.11 |
| CK_2 | 64,996,430 | 57,013,086 | 96.75 | 88.6 | 46.2 | | 74.95 |
| CK_3 | 64,996,844 | 56,494,272 | 96.75 | 88.43 | 45.9 | | 74.4 |
| L1_1 | 64,996,852 | 56,146,354 | 96.36 | 87.66 | 45.3 | | 74.73 |
| L1_2 | 63,240,188 | 56,356,942 | 96.36 | 87.53 | 45.6 | | 74.78 |
| L1_3 | 63,240,210 | 57,268,148 | 96.56 | 88.03 | 45.4 | | 74.9 |
| L2_1 | 64,995,946 | 57,175,440 | 96.69 | 88.48 | 45.4 | | 74.15 |
| L2_2 | 63,239,122 | 55,126,478 | 96.66 | 88.44 | 45.5 | | 74.21 |
| L2_3 | 64,995,708 | 56,752,882 | 96.78 | 88.67 | 46.1 | | 74.58 |
